# Comorbidities and Symptomatology of SARS-CoV-2 (Severe Acute Respiratory Syndrome Coronavirus 2)-Related Myocarditis and SARS-CoV-2 Vaccine-Related Myocarditis: A Review

**DOI:** 10.7759/cureus.24084

**Published:** 2022-04-12

**Authors:** Sarah Cushion, Vania Arboleda, Yousef Hasanain, Michelle Demory Beckler, Patrick Hardigan, Marc M Kesselman

**Affiliations:** 1 Medicine, Nova Southeastern University Dr. Kiran C. Patel College of Osteopathic Medicine, Davie, USA; 2 Osteopathic Medicine, Nova Southeastern University Dr. Kiran C. Patel College of Osteopathic Medicine, Fort Lauderdale, USA; 3 Osteopathic Medicine, Nova Southeastern University Dr. Kiran C. Patel College of Osteopathic Medicine, Davie, USA; 4 Microbiology and Immunology, Nova Southeastern University Dr. Kiran C. Patel College of Allopathic Medicine, Fort Lauderdale, USA; 5 Health Professions Division, Nova Southeastern University Dr. Kiran C. Patel College of Allopathic Medicine, Davie, USA; 6 Rheumatology, Nova Southeastern University Dr. Kiran C. Patel College of Osteopathic Medicine, Davie, USA

**Keywords:** covid-19 virus vaccine related myocarditis, covid-19 virus related myocarditis, myocarditis, sars-cov-2, covid-19

## Abstract

Myocarditis is an inflammatory disease of the heart muscle, with manifestations that include myocardial infarction, arrhythmia, and even sudden death. The primary etiology of myocarditis is a viral infection, with studies demonstrating that infection with severe acute respiratory syndrome coronavirus 2 (SARS-CoV-2) can lead to myocarditis. This enzyme is involved in many body tissues, including the gastrointestinal system and the cardiac system. This enzyme is responsible for converting angiotensin I to angiotensin II in the renin-angiotensin system of our body. This review aims to characterize the symptomatology and comorbidities of males, females, and pediatric patients who developed the SARS-CoV-2-related myocarditis (SARS-CoV-2RM) or the SARS-CoV-2 vaccine-related myocarditis (SARS-CoV-2VRM). From July 10 to July 20, 2021, a PubMed database search for “SARS CoV-2 Related Myocarditis” was conducted. From July 21 to July 30*,* 2021, the search for “SARS CoV-2 Vaccine Related Myocarditis” was conducted. The search completed was specific for title/abstract fields using keywords “Covid-19” AND “Myocarditis” AND “Vaccine” and specifying “Males” or “Females”, respectively. Inclusion criteria included articles discussing comorbidities and symptomatology. Exclusion criteria included autopsy/postmortem reports, letters to the editor, retrospective studies, and observational studies. In the end, 49 articles were found and included in this review. We found that 27 of 40 pediatric patients with SARS-CoV-2RM presented with gastrointestinal symptoms, and 12 of 40 pediatric patients had no comorbidities. In female cases, eight of 12 patients with SARS-CoV-2RM presented with noncardiac symptoms, and only four of 12 had comorbidities such as asthma, diabetes, and obesity. In male patients with SARS-CoV-2RM, 10 of 12 presented with respiratory and/or cardiac symptoms, and seven of 12 had cardiac and/or diabetic comorbidities. Furthermore, 22 of 31 male patients with SARS-CoV-2VRM presented with chest pain with no previous comorbidities; four of six females with SARS-CoV-2VRM presented with chest pain, and three of six females had no comorbidities; and seven of 11 pediatric patients with SARS-CoV-2VRM had no comorbidities, but 11 of 11 pediatric patients presented with chest pain. In conclusion, males, females, and pediatric patients with previous SARS-CoV-2VRM showed mostly chest pain with no comorbidities. Males presenting with SARS-CoV-2RM showed mostly respiratory and cardiac symptoms with cardiac and diabetic comorbidities. Females with SARS-CoV-2RM described various symptoms from flu-like, respiratory, to cardiac and had no previous comorbidities. The bulk of pediatric patients with SARS-CoV-2RM mainly presented with GI symptoms and no past comorbidities. More studies are needed to determine the clinical presentation and risk factors that lead to SARS-CoV-2RM and SARS-CoV-2VRM.

## Introduction and background

Myocarditis (MC) is classified as a heart muscle disease characterized by lymphocyte and macrophage infiltration into the heart, leading to myocardial injury without an ischemic cause [1]. The primary etiology of MC in the United States and other parts of the world is viral infection [2]. Other less frequently encountered etiologies include, but are not limited to, bacteria, fungi, hypersensitivity reactions to drugs, giant-cell myocarditis, or sarcoidosis [2]. MC presents in all age ranges and with various symptoms, ranging from mild dyspnea and palpitations that may resolve without specific therapy to complex cardiogenic shock and even sudden death [3]. This review will focus on the acute presentation of myocarditis (AMC) in women, men, and pediatric patients. AMC can last weeks to a couple of months and typically presents as a nonischemic dilated cardiomyopathy [4]. However, AMC can also present in a wide range of subclinical symptoms from new-onset atrial or ventricular arrhythmias, to complete heart block, or to acute myocardial infarction-like syndrome [4]. This literature review will highlight the known presentation of acute myocarditis in relation to the severe acute respiratory syndrome coronavirus 2 (SARS-CoV-2) viral infection and the SARS CoV-2 viral vaccines. 

Pathophysiology

The pathophysiology of SARS-CoV-2-related myocarditis (SARS-CoV-2RM) is still under question. One possible pathway is immune-mediated, involving both the innate and acquired immune responses. Depending on the host's immunity strength, the immune system may cause an inflammatory response leading to dilated cardiomyopathy. This inflammatory response can be mediated by several types of immune cells including T lymphocytes, neutrophils, dendritic cells, and macrophages [1].

In the case of SARS-CoV-2 directly invading myocardial tissue, the proposed pathophysiology of viral myocarditis suggests that the spike protein plays a key role in its ability to enter host cardiac cells. Viral entry is thought to occur as a result of binding of the viral spike protein to the host angiotensin-converting enzyme 2 receptor (ACE2R) [1]. The ACE2R is found in respiratory tissue on ciliated columnar epithelial cells and type II pneumocytes. Furthermore, ACE2R is also found within cardiomyocytes and is upregulated during heart failure. Because of this expression, it seems extremely plausible that SARS-CoV-2 may infect heart tissue directly via this receptor. SARS-CoV-2 viral proteins may send signals for cellular apoptosis and necrosis, leading to direct myocardial injury and infection [5]. Once heart cells are infected, antigen-presenting cells, dendritic cells, and macrophages present antigenic peptides to naive T lymphocytes, both in peripheral lymphoid organs and in the myocardium itself because certain cytokines produced by this tissue can recruit naive T lymphocytes [1,5-7]. Hepatocyte growth factor (HGF) is a chemoattractant produced by the myocardium, and it can lead to T-lymphocyte priming and recruitment during inflammation. HGF instructs T-lymphocyte cardiotropism with a signature, “c-Met+CCR4+CXCR3+.” c-Met is a tyrosine-protein kinase Met or hepatocyte growth factor receptor (HGFR) protein encoded by MET gene. CCR4 is part of the chemokine receptor family that is expressed on cells involved in the inflammatory processes such as basophils, eosinophils, and lymphocytes. CXCR3+ is a chemokine of the CXC chemokine class. An example of the CXC family includes interleukin (IL)-8 that specifically induces neutrophils and triggers further inflammation. c-Met recruits T cells by inducing autocrine chemokine receptor CCR5 ligand release, while CCR4 and CXCR3 also aid in recruitment [6-7]. Cluster of differentiation (CD) is used to classify surface antigens on leukocytes. Effector CD8+ and CD4+ T lymphocytes can target myocardial tissue through several mechanisms. These include direct killing of SARS-CoV-2-infected myocytes by effector CD8+ T lymphocytes and secretion of proinflammatory cytokines by CD4+ T lymphocytes [6]. These cytokines appear to mediate the inflammatory response responsible for disease by recruiting macrophages and neutrophils to the cardiac sites. In extreme cases, these cytokines induce a feed-forward loop, resulting in cytokine storm syndrome. From activation of effector CD8+ and CD4+ T lymphocytes, macrophages, and dendritic cells, ultimately, inflammation of the myocardium can result in myocarditis [1].

## Review

Methods

The search for case reports was done through a PubMed database search. The search for “SARS CoV-2 Related Myocarditis” was conducted from July 10 to July 20, 2021. The search for “SARS CoV-2 Vaccine Related Myocarditis” was conducted from July 21 to July 30, 2021.

Search

**Figure 1:**
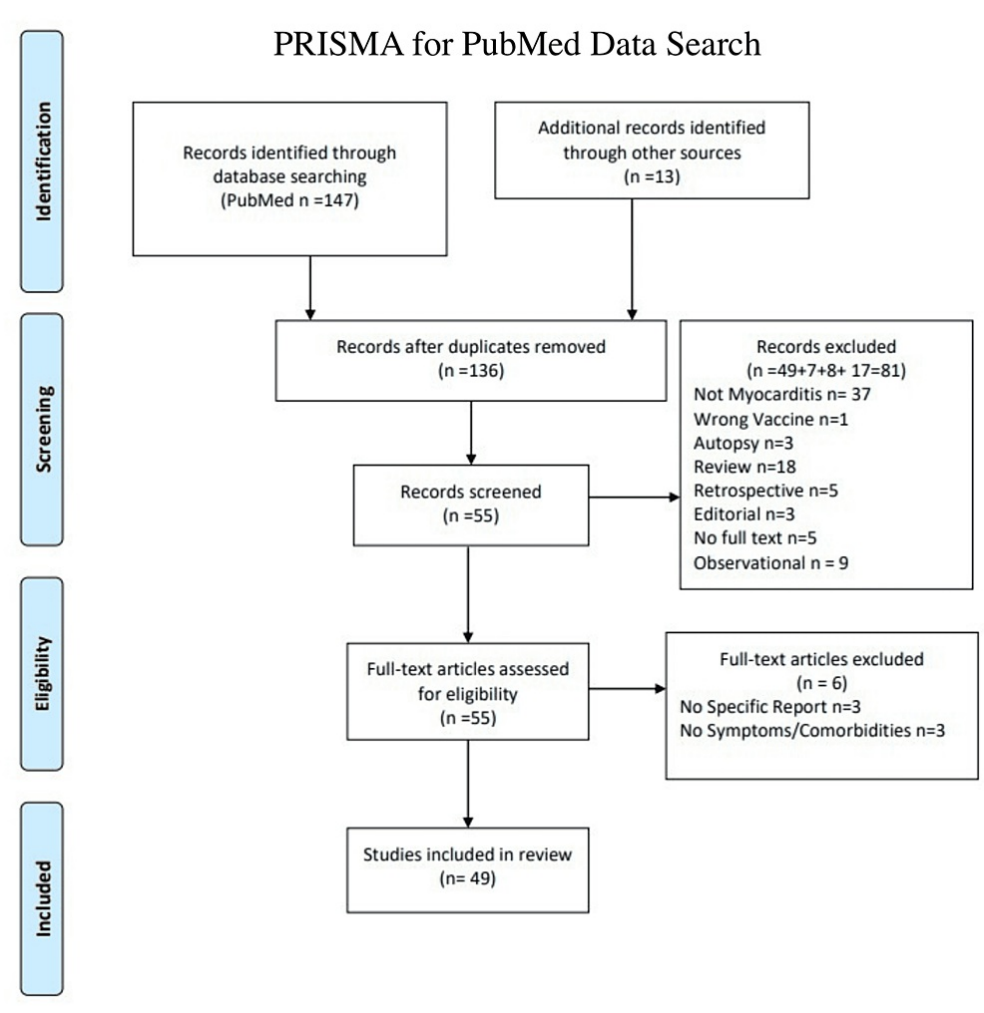
Preferred Reporting Items for Systematic Reviews and Meta-Analyses (PRISMA) Chart

The searches completed were specified for the title/abstract fields. The search flow chart is illustrated in Figure 1. One search for SARS-CoV-2 vaccine-related myocarditis for adult males was “Covid-19” AND “Myocarditis” AND “Vaccine” AND “males.” One search for SARS-CoV-2 vaccine-related myocarditis for adult females was “Covid-19” AND “Myocarditis” AND “Vaccine” AND “females.” One search for SARS-CoV-2 vaccine-related myocarditis for pediatric cases was “Covid-19” AND “Myocarditis” AND “Vaccine” AND Pediatrics.” One search for SARS-CoV-2-related myocarditis for adult males was “Covid-19” AND “Myocarditis” AND “males.” One search for SARS-CoV-2-related myocarditis for adult females was “Covid-19” AND “Myocarditis” AND “females.” To note, some female cases were found in the male search since “male” is in the word “female.”

Inclusion Criteria

The inclusion criteria included the articles specifically discussing SARS-CoV-2-related myocarditis and SARS-CoV-2 vaccine-related myocarditis as defined in this review. Additionally, articles discussing both comorbidities and symptomatology for the population were also included. The exception to this was a pediatric article, “Acute myocarditis and multisystem inflammatory emerging disease following SARS-CoV-2 infection in critically ill children” [13]. This paper did not discuss comorbidities and only discussed the symptomatology of pediatric patients in the SARS-CoV-2-related myocarditis population. The reason for this exception was due to the limited data; during the search, we decided that it would be beneficial to include this report that had described 20 patients.

Exclusion Criteria

The exclusion criteria included: the article was not myocarditis related, the incorrect vaccine was mentioned, any autopsy/postmortem reports, retrospective, letters to the editor, no full text found, observational studies, no specific report mentioned, and no symptoms or comorbidities mentioned. To note, of the reviews found, the references from those reviews were used for individual studies and those are included in the “Additional records identified through other sources” section in the Preferred Reporting Items for Systematic Reviews and Meta-Analyses (PRISMA) chart (Figure 1). 

Findings

With all the searches combined, 147 studies were identified in the PubMed search. Due to overlap in terminology, 24 duplicates were removed. Additionally, 13 references were found cited within the resulting articles [1-53]. Using the exclusion criteria, 81 records were removed. Forty-nine records from the pediatrics population with both SARS-CoV-2RM and SARS-CoV-2VRM were removed. Fifteen records from the male population with SARS-CoV-2VRM and SARS-CoV-2RM were removed. Seventeen records were removed from the female population with SARS-CoV-2RM and SARS-CoV-2VRM. After full-text review, six articles were excluded due to not finding specific reports or symptomatology. After screening and exclusions, 49 articles in total were found among all the categories for use.

SARS-CoV-2-related myocarditis (SARS-COV-2RM)

This review found a total of 44 cases reporting on SARS-CoV-2RM comorbidities: a total of 20 pediatric patients, 12 male patients, and 12 female patients were reported. By viewing a patient’s comorbidities and symptomatology, clinicians can diagnose and treat earlier, leading to reduced mortality. Typically, comorbidities can predispose a patient to certain diseases. This review will focus on the comorbidities and symptomatology of SARS-CoV-2 in these three populations [1,8-42].

Comorbidities

**Figure 2:**
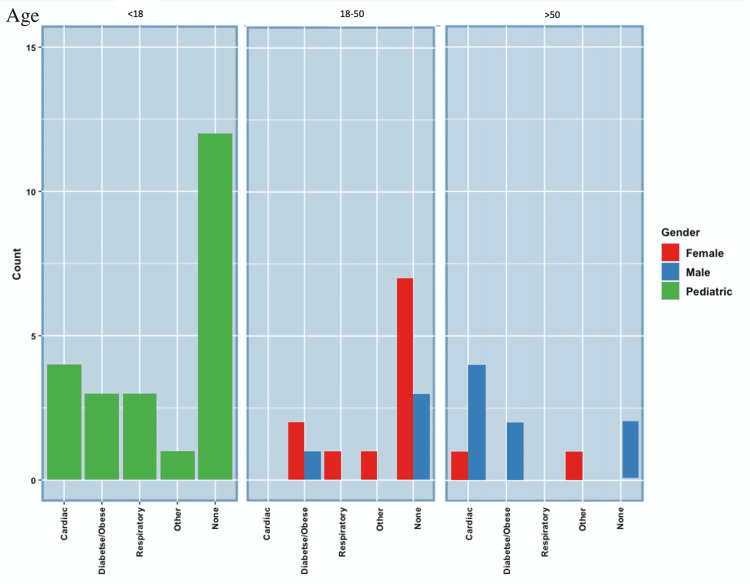
SARS-CoV-2-Related Myocarditis Comorbidities SARS-CoV-2: severe acute respiratory syndrome coronavirus 2.

For pediatric patients with SARS-CoV-2RM, eight of 20 presented with previous comorbidities: two of 20 presented with atrial septal defects, three of 20 presented with asthma, two of 20 presented with obesity, and one presented with autism. The other 12 pediatric patients with SARS-CoV-2RM had no comorbidities [8-20]. For the adult population, seven of 12 male patients with SARS-CoV-2RM showed cardiac or respiratory comorbidities, including arrhythmias, hypertension, asthma, and chronic obstructive pulmonary disease (COPD). The other five male SARS-CoV-2RM patients had no comorbidities [21-31]. Of the five males with no comorbidities, two were over the age of 50 and three were under the age of 50. On the other hand, eight of 12 female SARS-CoV-2RM patients presented with no comorbidities. For the female patients with SARS-CoV-2RM comorbidities, two of 12 presented with obesity, two of 12 presented with diabetes, one of 12 presented with asthma, one of 12 presented with atrial fibrillation, and only one presented with multiple sclerosis [32-42]. Seven of 12 females with no comorbidities were all under the age of 50. Figure 2 and Table 1 summarize the comorbidities in pediatric, male, and female SARS-CoV-2RM patients [1,8-42].

**Table 1:** SARS-CoV-2-Related Myocarditis (SARS-CoV-2RM) Comorbidities and Symptomatology and SARS-CoV-2 Vaccine-Related Myocarditis (SARS-CoV-2VRM) Comorbidities and Symptomatology SARS-CoV-2: severe acute respiratory syndrome coronavirus 2.

SARS-CoV-2RM (SARS-CoV-2RM)						SARS-CoV-2 (SARS-CoV-2VRM)					
Comorbidities						Comorbidities					
Gender	Male		Female		Pediatrics	Gender	Male		Female		Pediatrics
Number of cases	n=12		n=12		n=20	Number of cases	n=11		n=6		n=11
Age	18-50	>50	18-50	>50	<18	Age	18-50	>50	18-50	50	<18
Respiratory			1		3	Respiratory	2		1		
Cardiac		4		1	4	Cardiac				1	1
Diabetic/obese	1	2	2		3	Diabetic/obese					1
Other			1	1	1	Other	1			1	2
None	3	2	7		12	None	2	7		3	7
Presenting symptoms						Presenting symptoms					
Gender	Male		Female		Pediatrics	Gender	Male		Female		Pediatrics
Number of cases	N=12		N=12		N=40	Number of cases	n=31		n=6		n=11
Age	18-50	>50	18-50	>50	<18	Age	18-50	>50	18-50	>50	<18
Flu-like symptoms	2	1	3	1	8	Flu-like symptoms	1				
Cardiac symptoms		4	3	1	4	Cardiac symptoms	30		3	1	11
GI symptoms		1	3		27	GI symptoms					1
Respiratory symptoms	2	4	2		8	Respiratory symptoms				1	
Other/general		1		1	2	Other/general				1	

Symptomatology 

**Figure 3:**
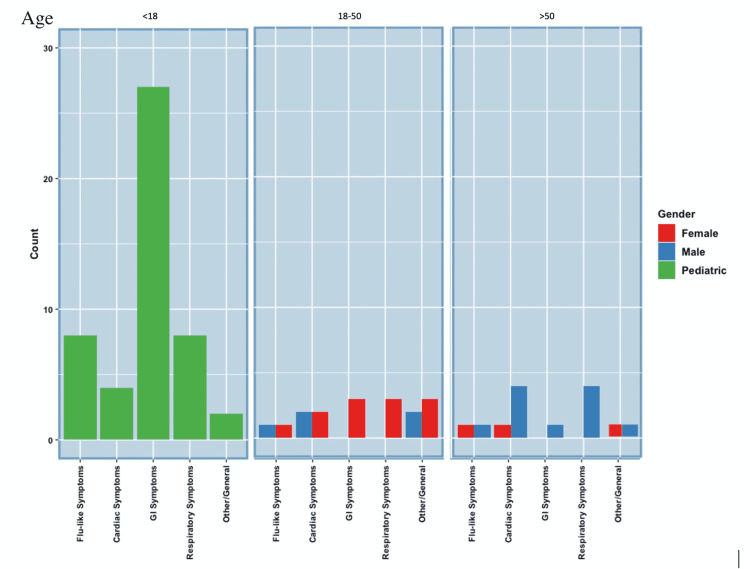
SARS-CoV-2-Related Myocarditis Symptomatology SARS-CoV-2: severe acute respiratory syndrome coronavirus 2.

In SARS-CoV-2RM comorbidity research, only 20 cases were found in the pediatric population to discuss comorbidities; however, a total of 40 cases, including 20 from the comorbidity section, described symptomatology in the pediatric population with SARS-CoV-2RM. The chief complaint for 27 of 40 pediatric patients with SARS-CoV-2RM was of GI origin, including abdominal pain, nausea, vomiting, and diarrhea. Eight of 40 presented with flu-like symptoms, four of 40 presented with cardiac, eight of 40 presented with respiratory, and two of 40 presented with nonspecific symptoms such as fatigue [8-20]. For the adult population with SARS-CoV-2RM, only one of 12 male patients with SARS-CoV-2RM presented with no symptoms. For the other male patients with SARS-CoV-2RM, three of 12 presented with flu-like symptoms, one of 12 presented with GI symptoms such as abdominal pain or nausea, and 10 of 12 presented with cardiac symptoms, such as chest pain and palpitations, or respiratory comorbidities, such as dyspnea [21-31]. To note, eight of 12 male patients presenting with cardiac symptoms and respiratory symptoms were all over the age of 50. However, it is also noted that more male cases over the age of 50 were found than those under the age of 50. For the female SARS-CoV-2RM patients, four of 12 presented with cardiac symptoms such as chest pain or palpitations. Two of 12 presented with respiratory symptoms such as dyspnea. Three of 12 presented with GI symptoms such as abdominal pain, nausea, vomiting, or diarrhea. Four of 12 presented with flu-like symptoms such as congestion, sore throat, and fever, and only one female SARS-CoV-2RM patient presented with a chief complaint of fatigue [32-42]. Of the female population, five of 12 patients presenting with cardiac or respiratory symptoms were under the age of 50 and one of 12 presenting with cardiac symptoms was over the age of 50. Figure 3 and Table 1 summarize the symptomatology of pediatric, male, and female patients with SARS-CoV-2RM [1,8-42].

SARS-CoV-2 vaccine-related myocarditis (SARS-CoV-2VRM)

The vaccines considered in this review were Moderna vaccine mRNA-1273, Pfizer-BioNTech COVID-19 vaccine BNY162b2, Janssen/Johnson & Johnson (Ad26.COV2.S), and CoronaVac [43-55]. This review found a total of 28 cases reporting on SARS-COV-2VRM that discussed comorbidities: 11 reported on pediatric patients [43-45], 11 on male patients [46-53], and six on female patients [54-55]. No male patients over the age of 50 were found to have comorbidities in this part of the literature review. This review found a total of 48 cases reporting on SARS-CoV-2VRM that discussed symptomatology: 11 in pediatric patients, 31 in male patients, and six in female patients. This section will focus on the comorbidities and symptomatology of SARS-CoV-2VRM in these three populations. 

Comorbidities

**Figure 4:**
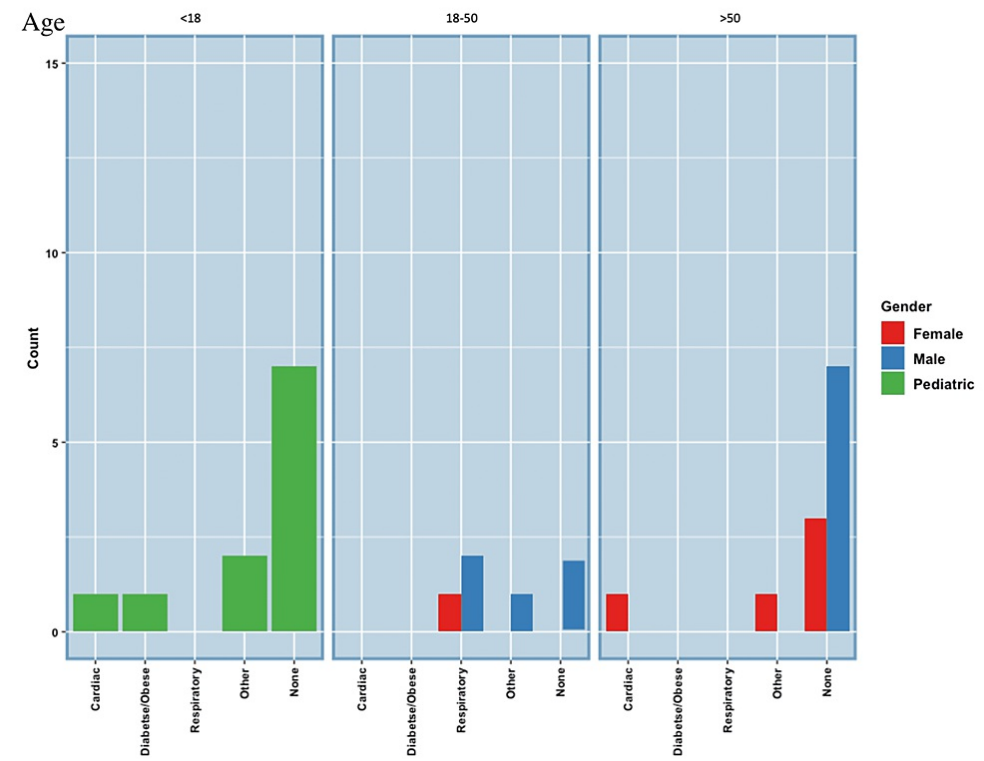
SARS-CoV-2VRM Comorbidities SARS-CoV-2VRM: severe acute respiratory syndrome coronavirus 2 vaccine-related myocarditis.

In the pediatric SARS-CoV-2VRM patients, seven of 11 presented with no comorbidities. The other four pediatric SARS-CoV-2VRM patients presented with comorbidities, one presented with obesity, insulin resistance, and dyslipidemia, one presented with Marfan syndrome and aortic root dilation, one presented with vitiligo, and one presented with perimyocarditis [43-45]. For the adult population, nine of 11 male SARS-CoV-2VRM patients had no comorbidities. Of the other two male SARS-CoV-2VRM patients with comorbidities, both presented with asthma, and one presented with obstructive sleep apnea [46-53]. It is noted that no males over the age of 50 had comorbidities. For the female SARS-CoV-2VRM patients, three of six females had comorbidities, one presented with multiple sclerosis, one presented with hypercholesterolemia, hypertension, and smoking, and one presented with asthma. The other three female SARS-CoV-2VRM patients had no comorbidities [47,49,54-55]. Figure 4 and Table 1 summarize the comorbidities in pediatric, male, and female SARS-CoV-2VRM patients. 

Symptomatology 

**Figure 5:**
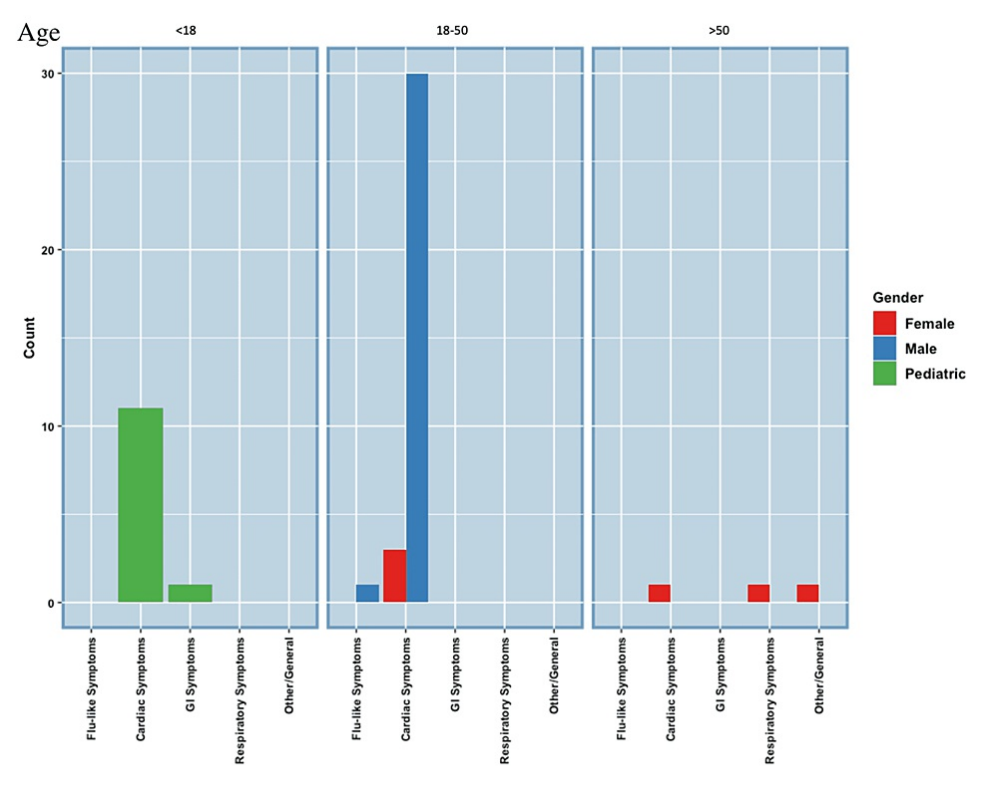
SARS-CoV-2VRM Symptomatology SARS-CoV-2VRM: severe acute respiratory syndrome coronavirus 2 vaccine-related myocarditis

All 11 pediatric patients with SARS-CoV-2VRM presented with chest pain [43-45]. One pediatric SARS-CoV-2VRM patient with chest pain also presented with abdominal pain [45]. For the adult population, not 11 but 31 cases were found reporting on the symptoms of SARS-CoV-2VRM in male patients. Thirty of 31 male SARS-CoV-2VRM patients presented with cardiac symptoms such as chest pain or palpitations [46-53]. Seven male comorbidity cases that were over the age of 50 did not mention symptomatology and, therefore, were not included in the symptomatology (Figure 5). For the female SARS-CoV-2VRM patients, four of six presented with cardiac symptoms, the most common being chest pain [47,49,54-55]. One patient presented with type 1 Kounis syndrome which is a result of mast cell and platelet activation causing an allergic reaction. This syndrome leads to coronary spasm which involves palpitations, flushing, dyspnea, and chest pain [55]. One of six presented with shortness of breath. Figure 5 and Table 1 summarize the symptomatology of pediatric, male, and female patients with SARS-CoV-2VRM.

Discussion 

This review sought to determine the comorbidities that present in patients with SARS-CoV-2-related myocarditis (SARS-CoV-2RM) or SARS-CoV-2 vaccine-related myocarditis (SARS-CoV-2VRM). Male, female, and pediatric populations were examined in search of commonalities in presenting symptoms and associated comorbidities. The aim of this study was to illustrate patterns of presentation and comorbidities so that clinicians may be able to better identify, diagnose, and care for patients with SARS-CoV-2-related myocarditis and SARS-CoV-2 vaccine-related myocarditis.

Presenting Symptomatology

Presenting symptomatology in SARS-CoV-2RM cases across male, female, and pediatric populations was examined. The most remarkable finding was the link between GI symptoms and SARS-CoV-2RM among the pediatric population. In patients under the age of 18 (regardless of gender), we found that 27 out of 40 total cases presented with GI symptoms. Of the 27 cases, 25 had abdominal pain, two had nausea and vomiting, and three had diarrhea [8-20]. GI symptoms were not significant in the male and female populations [1,21-42]. This is a significant finding as it demonstrates a clear pattern of clinical presentation among pediatric patients. GI symptoms in pediatric patients should raise the index of suspicion in clinicians, parents, teachers, and caregivers, of the root cause being SARS-CoV-2-related myocarditis [8,12,15].

In cases of SARS-CoV-2VRM, there was a strong predilection in men, women, and pediatrics for presenting with cardiac symptomatology which included chest pain (left side, retrosternal, substernal), palpitations, and dyspnea [43-55]. Of the 31 male patients examined, 30 presented with cardiac symptoms. Of the 11 pediatric patients examined, all 11 patients presented with cardiac symptoms. This shows a clear distinction between the presentation of SARS-CoV-2RM and SARS-CoV-2VRM. Whereas SARS-CoV-2RM may present with a wider variety of symptoms ranging from cardiac to gastrointestinal [8-42], SARS-CoV-2VRM presentation is likely to be cardiac in nature [43-55]. In general, cases of SARS-CoV-2VRM appeared less frequently in females; therefore, there were fewer cases to draw data from. Of the six female cases that were reviewed, four of six presented with cardiac symptoms [36,44,50,55]. Further research is recommended in male, female, and pediatric patients to determine the typical presentation of SARS-CoV-2VRM.

Comorbidities

The presence of other medical conditions, such as Marfan syndrome, multiple sclerosis, asthma, hypertension, hypercholesterolemia, and aortic root dilation, may act as a contributing factor to the development of SARS-CoV-2RM or SARS-CoV-2VRM [16,18,20,30,45,54]. In this study, we particularly focused on the existence of respiratory, cardiac, diabetic/obesity-related comorbidities [8,16,36]. Twelve males with SARS-CoV-2RM were examined, four of 12 presented with cardiac comorbidities, and three of 12 presented with diabetic/obese comorbidities. Twelve females were examined, one of 12 presented with respiratory comorbidities, one of 12 presented with cardiac comorbidities, and two of 12 presented with diabetic/obese comorbidities. Eight females presented with no comorbidities. Twenty pediatric cases were examined, four of 20 presented with cardiac comorbidities [8,42,45]. The main comorbidities found in all populations were cardiac. However, the vast majority of cases presented with no comorbidities. This further indicates that patients without cardiac comorbidities were not excluded from potential cardiac effects [17,36,44,50].

In cases of SARS-CoV-2VRM, of 11 male cases examined, two of 11 presented with respiratory comorbidities, and seven of 11 presented with no comorbidities. Of six female cases examined, one presented with multiple sclerosis, one presented with respiratory comorbidities, one presented with cardiac comorbidities, and three presented with no comorbidities. Eleven pediatric cases were examined, one of 11 presented with cardiac comorbidities, one of 11 presented with diabetic/obese, and seven of 11 presented with no comorbidities. Overall, most cases presented with no comorbidities. This further indicates the possibility that myocarditis is a result of the host’s immune response to vaccination. Pediatric cases had developed a stronger response to the vaccine after the second dose than the adult population and it was ruled out that patients had developed a respiratory viral illness. This indicates that there were no other contributing factors in these SARS-CoV-2VRM patients [50]. 

Further Investigation and Limitations

Potential limitations of this research include the low number of case reports particularly in the female SARS-CoV-2VRM population. We recommend further investigation among all populations as more cases of SARS-CoV-2RM and SARS-CoV-2VRM arise. This research also did not account for the potential of overlapping comorbidities in a single patient and how this overlap could affect the patient's presentation. Future studies should further focus on overlapping comorbidities and any patterns that may emerge in clinical presentation. The possibility that certain set of comorbidities predisposes patients to developing myocarditis following either vaccination of COVID-19 infection is still to be determined since this review did not specifically line up comorbidities of one patient to the same patient’s symptomatology. Lastly, if the female cases are lower than the other two populations based on female physiology, then the lower number of cases in females may suggest a cardioprotective feature in female patients [56-57]. Additionally, we recommend further investigation regarding the lack of clinical feature awareness in female cardiac patients. 

## Conclusions

The full effects of the SARS-CoV-2 virus and the SARS-CoV-2 vaccine on the human heart remain a question. However, with further research, clinicians can begin to develop a higher suspicion of myocarditis in these various populations who present without classic cardiac symptoms. The SARS-CoV-2RM had more variation of symptoms including fatigue, flu-like symptoms, and gastrointestinal symptoms, whereas the SARS-CoV-2VRM population presented with mostly cardiac symptoms, specifically chest pain. Based on the results, we recommend further research on these groups, particularly the female SARS-CoV-2VRM group, as the results were limited in this review. As of now, it is suggested that patient comorbidities and presentation be considered in each patient who presents with SARS-CoV-2 virus or SARS-CoV-2 vaccine adverse effects. The long-term implications are still debated. The treatments for these patients should follow the current protocol and recommendations for each age group.
